# Test allocation based on risk of infection from first and second order contact tracing

**DOI:** 10.1371/journal.pone.0320291

**Published:** 2025-04-07

**Authors:** Soler Gabriela Bayolo, Felipe Miraine Dávila, Ghislaine Gayraud

**Affiliations:** 1 LMAC (Laboratory ofApplied Mathematics of Compiègne), Université de technologie de Compiègne,Compiègne, France; University of Zaragoza, SPAIN

## Abstract

Strategies such as testing, contact tracing, and quarantine have been proven to be essential mechanisms to mitigate the propagation of infectious diseases. However, when an epidemic spreads rapidly and/or the resources to contain it are limited (e.g., not enough tests available on a daily basis), to test and quarantine all the contacts of detected individuals is impracticable. In this direction, we propose a method to compute the individual risk of infection over time, based on the partial observation of the epidemic spreading through the population contact network. We define the risk of individuals as their probability of getting infected from any of the possible chains of transmission up to length-two, originating from recently detected individuals. Ranking individuals according to their risk of infection can serve as a decision-making tool to prioritise testing, quarantine, or other preventive measures. We evaluate interventions based on our risk ranking through simulations using a fairly realistic agent-based model calibrated for COVID-19 epidemic outbreak. We consider different scenarios to study the role of key quantities such as the number of daily available tests, the contact tracing time-window, the transmission probability per contact (constant versus depending on multiple factors), and the age since infection (for varying infectiousness). We find that, when there is a limited number of daily tests available, our method is capable of mitigating the propagation more efficiently than some other approaches in the recent literature on the subject. A crucial aspect of our method is that we provide an explicit formula for the risk, avoiding the large number of iterations required to achieve convergence for the algorithms proposed in the literature. Furthermore, neither the entire contact network nor a centralised setup is required. These characteristics are essential for the practical implementation using contact tracing applications.

## Introduction

In the context of epidemics, contact tracing is the process of identifying individuals who have been in contact with other individuals diagnosed with a transmissible disease. The relevant contacts are those that would allow the transmission to happen, which depends on the mode of transmission of the disease, and requires the detected individual to be infectious at the time of the encounter. Together with strategies such as testing and quarantining, contact tracing has been shown to be an essential mechanism in order to mitigate the spread of a disease, allowing to contain and delay outbreaks, see [1]. Ideally, detected individuals (those who receive a positive test result) are quarantined and their contacts are identified and tested as well. However, these interventions have an economic and social cost, and a scenario where all the contacts of detected individuals are tested is not realistic for diseases that start spreading quickly in the population (outbreak). Hence, when the resources are limited (e.g., amount of daily available tests), the question of how to cleverly allocate them to the population arises.

In this direction, we propose a method to compute the risk of infection of individuals in the population over time, based on the partial observation of the epidemic spreading through the population contact network. The risk of each individual is defined as her/his (marginal) probability of infection conditionally on the observed variables in the recent past, and the higher-risk individuals can get notified to be tested, quarantined, or applied any other preventive measures. Thus, the quantification of the infection risk is proposed here as a tool to allocate the available resources more rationally than just randomly. Similar intervention approaches have been shown to have a positive impact on mitigating epidemics, as seen in [2–12].

To be more precise about our approach, we consider an agent-based model on a fixed-size population where individuals admit a set of (discrete) characteristics influencing the transmission of the disease. The pairwise contacts between individuals are described by a *dynamical network* model, in which some connections are deleted and some others are created while time evolves. Then, we depict the disease spread in the population contact network by a stochastic *Susceptible-Infectious-Removed* (SIR) dynamic (including more than three classes), meaning that contagion can only happen (with a certain probability) when an infectious individual is connected with a susceptible one by an edge in this contact network. In particular, we consider a non-Markovian dynamic since the infection probability per interaction depends on the date of infection of the source. This probability is also a function of the previously mentioned characteristics of both individuals and the context of the interaction (e.g., in the household, workplace, or random).

Given this dynamical network model and the propagation process on this network, we suppose that the infectious statuses of individuals are (partially) observed through testing, as well as the underlying contact network, the factors having an impact on the transmission for the set of tested individuals, and their direct and secondary contacts. Actually, we consider *at risk* not only the first-degree contacts of detected individuals (1^∘^ contacts) but also their subsequent contacts ($2^{\circ }$ contacts). In the sequel, to distinguish the different groups of individuals under consideration, we call *index cases* the detected individuals while they are infectious, *$1^{\circ }$ contacts* the individuals who are not detected but interacted with index cases while the latter were infectious, and, *$2^{\circ }$ contacts* the individuals who are not detected but interacted with $1^{\circ }$ contacts after the latter were in contact with index cases. In addition, *$1^{\circ }$ interaction* and *$2^{\circ }$ interaction* refer to the risky encounter between index cases and $1^{\circ }$ contacts, and between $1^{\circ }$ and $2^{\circ }$ contacts respectively.

In real situations, the information about the tested individuals and their $1^{\circ }$ and $2^{\circ }$ contacts is provided either by individuals themselves, through manual contact tracing (MCT), or by a digital contact tracing (DCT), and is not necessarily centralized in the digital case. The advantages of extending the contact tracing strategies up to $2^{\circ }$ contacts have been highlighted in the recent literature, see [7,13,14]. Then, to compute the risk, we consider a rather general probability of transmission per interaction, depending on the observed attributes of both individuals, characteristics of the interaction, and the infection time of the source (which is unknown and estimated from the observations). Finally, we compute the probability for each individual at risk of having been infected by one of the individuals detected in the previous days (fixed time window), through chains of transmission of length one or two. It is worth noticing that we compute this marginal probability by summing over all the possible paths of transmission, providing explicit formulas, and avoiding independence assumptions and the *cycling back* phenomenon described in S2 appendix.

Finally, we propose and simulate the following mitigation strategy: every day the probability of being infected is computed for the individuals at risk, and a fixed number of the highest-ranked individuals are tested; the newly detected individuals are put in quarantine the day after, and the process is repeated each day during an intervention period. In parallel of the detection by risk, symptomatic individuals are tested with a fixed probability per day since the beginning of their symptoms, the ones detected are quarantined, and their contacts are traced as described before. We evaluate this intervention through simulations in a series of different scenarios, where we study the impact of some of the parameters in the model such as the number of daily available tests, the time-frame for the time of infection and the proportion of detection of symptomatic individuals. We also study the influence of the probability function of transmission per interaction and different ways to estimate the time of infection of the source. We further investigate the influence of the mean detection time for individuals who develop symptoms, test sensitivity and specificity, and the quarantine adoption fraction. Additionally, we examine the role of super-spreaders within our model. We found that, in most cases, our test allocation method is capable of mitigating the propagation of the disease considerably faster than randomly selecting (RS) individuals to get tested, or the usual contact tracing (CT, i.e. ranking according to the number of interactions with detected individuals). Moreover, we found that with fewer daily available tests, our risk ranking is more efficient than an equivalent setting where the probabilities are computed under the mean-field (MF) hypothesis, see [2].

In the recent literature, there are many research works that study the effect of contact tracing combined with treatment and/or quarantine, as non-pharmaceutical interventions for infectious disease mitigation and control, see [15]. The SARS-CoV-2 outbreak has considerably increased the scientific research motivation around strategies including contact tracing. In particular, DCT apps have attracted the attention of Public Health authorities and the scientific community as well, [16,17]. DCT apps allow to collect the information automatically, and provide fast processing times but also come with drawbacks regarding privacy and the data protection standards ruling in most countries, see [18]. This privacy protection issues have had to be carefully addressed in order to implement this type of strategies in practice. It is not our aim to discuss here how DCT apps should be implemented, however, it is worth noticing that the tracing of random interactions, included in our simulations, is only possible through DCT. The effectiveness of contact tracing interventions for the COVID-19 pandemic is studied in [19] and more recently in [17,20], providing reviews of what has been done on this topic based on empirical and simulated data.

Mathematical models play a crucial role in understanding the spread of epidemics, predicting their progression, guiding public health decision-making, and evaluating the effectiveness of intervention measures. While simpler models are easier to understand and implement, they often represent reality in a more abstract manner, capturing only essential features [21,22]. Among the most widely recognized families of epidemic models are the compartmental SI models, which describe epidemic spread within a homogeneous population by classifying individuals into groups based on their epidemic status [23].

To achieve greater realism, these compartmental models have been extended to agent-based models that account for the social structure and individual interaction patterns within a population. The pandemic caused by SARS-CoV-2 has significantly increased interest in these models, in particular for evaluating the impact of non-pharmaceutical intervention strategies such as quarantine, contact tracing, lockdown, and social distancing [24–26]. Agent-based models incorporating contact network structures further enhance realism by accounting for heterogeneities in social interactions, which are critical for understanding and mitigating the spread of infectious diseases. In this work, we adopt an agent-based modeling approach based on networks to provide a realistic framework for assessing our proposed contact tracing method.

Specifically, in our simulations we use the Oxford OpenABM-Covid19 model, which defines a contact network based on demographic characteristics of the UK population calibrated for the transmission of airborne diseases [27]. Furthermore, in this model, the spread of the COVID-19 epidemic follows an enriched SIR dynamic (11 possible disease statuses), stratified by age group and context of the interaction (e.g., household, workplace, random encounter). It also accounts for key epidemiological features of the COVID-19 outbreak. In particular, the model defines an infectiousness function that varies with time since infection and considers asymptomatic and presymptomatic states. By capturing essential aspects of real-world contact patterns and the epidemiology of COVID-19, this model provides a solid foundation for evaluating the proposed risk estimation method. It reflects not only the structural heterogeneity of populations, but also the dynamic and nuanced nature of epidemic transmission, making it highly suitable for our analysis.

Among the recent research studies looking at non-pharmaceutical interventions strategies, there are a few with the same aim as ours, that is, to integrate individual infection risk levels based on the observation of the interaction network and the test results in order to optimize the allocation of the available resources. In [7] and [4], the risk is computed using Monte Carlo methods. In [7], the authors estimate the individual infection probability up to $3^{\circ }$ contact tracing, arguing that it improves the detection of asymptomatic patients in diseases with a high percentage of them. Here, instead, we derive an explicit formula for these probabilities taking into account up to $2^{\circ }$ contacts, and hence avoiding the large computing power and the centralized information required by Monte Carlo methods. Other works such as [2] and [6] avoid the use of Monte Carlo methods by using the mean-field approximation to evaluate the individual risk of infection. However, the way the risk is propagated is “bidirectional,” meaning that it is not only “forward” in the direction of the transmission given the observations. Indeed, they suppose that individuals interchange their risk information at each time step if there is an edge between them, regardless of the previous path followed by the transmitted risk, falling sometimes in the above mentioned cycling back issue. Despite the similarities in the use of the MF hypothesis, it should be noticed that in [6] the network and propagation model are simpler than in [2]. The latter deals with more realistic models, including the OpenABM-Covid19 model used in our simulations. Moreover, in [2] a second method is developed, that estimates the individual infection risk as the posterior distribution conditional on the test observations through the Belief Propagation (BP) inference algorithms. Similar computations are achieved in [3] and [5] using Gibbs Sampling (GS) and Factorized Neighbors (FN) respectively. Another related methodology is presented in a series of works [9,10,12,28–32], where the authors achieve the risk computation using deep learning algorithms based on neural networks. It is also worth mentioning other machine learning approaches for risk estimation from DCT data that exploit different observations, such as Bluetooth energy measurements and exposure data, see [8,11].

A crucial aspect of our work is that we consider a rather realistic contact network and a detailed disease spread model, see [27]. Another significant aspect is our consideration of $2^{\circ }$ contact tracing, which provides a more accurate estimation of the risk compared to $1^{\circ }$ contact tracing. Although our approach can be extended to $3^{\circ }$ contacts and beyond, the calculations would get much heavier, and we argue that the gain in the effectiveness of the mitigation would not be significant, due to the uncertainty on the statuses of the intermediate individuals in the chains of transmission. One more core feature of our method is that to compute the risk, neither the whole contact network nor a centralized setup (contacts and individual information) is required. These characteristics are crucial for practical implementation using DCT applications, where the interchange of information between contacts across the entire network could be challenging due to privacy restrictions impacting a vast amount of personal data. These challenges can be exacerbated in centralized systems, see [5]. Furthermore, compared to the previously mentioned inference algorithms used to calculate the individual risk of being infected (i.e., BP, GS, FN), our risk calculation is simpler: while these algorithms integrate the observations at any time *t* by updating and re-propagating the risks step-by-step in a given time interval previous to *t* for every contact (up to any contact degree) of all the individuals in the population, we calculate directly the risk at *t* of individuals in contact (up to $2^{\circ }$) with someone detected by integrating the probability of any possible path of length up to 2 that might lead to the infection of these individuals. In this way, we avoid any cycling back phenomenon, and we do not need to update the risk of all individuals for every time step in the contact tracing time window, getting a very low level of messages interchange between individuals, see [5].

## Methods

We aim at defining a practical, realistic, and efficiently implemented risk-based dynamic detection process allowing us to identify the most likely infected individuals. To take into account the heterogeneity of disease transmissions, we consider that the probability of infection per interaction depends on individual attributes (e.g., age, healthy habits) and infectiousness of the source (e.g., day since infection, type of symptoms) of individuals in contact and the characteristics of the interaction (e.g., place, duration, distance, protective measure).

In addition, to provide a sharper and earlier detection process of the most likely infected individuals, not only the direct contacts of detected individuals are considered to be at risk, but also their subsequent contacts ($2^{\circ }$ contact tracing). Compared with the usual $1^{\circ }$ contact tracing, we expect to obtain a more accurate estimation of the risk of infection, and hence to detect more efficiently (in terms of the mitigation of the epidemic) individuals that are in general harder to detect due to the absence of symptoms (asymptomatic or pre-symptomatic individuals). It has become clear from numerous research studies that these latter individuals play an important role in SARS-CoV-2 transmission dynamics, see [13,14,33].

From a realistic point of view, one can estimate the individual risk of getting infected only from the observations (available information) that are provided by the tested individuals and their contacts through manual or digital CT. In view of all the above, our intervention approach relies on a dynamic risk evaluation for $1^{\circ }$ and $2^{\circ }$ contacts, and their risk is defined as the marginal conditional probability of getting infected given their past known $1^{\circ }$ and $2^{\circ }$ interactions, and the information provided by the tests.

In the following, we give a brief description of disease-spread models on social networks, we introduce some useful notations and finally, we define the risks of infection for $1^{\circ }$ and $2^{\circ }$ contacts.

### Disease spread model on social networks

We consider a population consisting of *N* (*N* ∈ *ℕ*) individuals that stays constant over time, so neither births, deaths nor migrations are taken into account. Notice that constant population size is a convenient assumption for the notation, but small variations in the population size do not influence the procedures described in the sequel. Let us denote by *V* = { 1 , … , *i* , … , *j* , … , *N* }  the population under consideration.

*Social structure model*

At any discrete time *t* (*t* ∈ *ℕ*), the social structure (interactions between individuals at *t*) is represented by an undirected graph $G_{t}=\left(V,E_{t}\right)$. We consider that $V=\{(i,a^{i}):i\in V\text{ and }a^{i}\in A\}$ corresponding to the set of vertices *V* (the individuals), supplemented by the set *A* of vertices attributes mentioned at the beginning of the section. Likewise, $E_{t}=\{(i,j,c_{t}^{ij}):(i,j)\in E_{t}\text{ and }c_{t}^{ij}\in C\}$ is the set of the edges $E_{t}$, describing the interactions at time *t* between the corresponding individuals, supplemented by *C*, the set of the characteristics of these interactions.

The time interval  [ 0 : *T* ]  corresponds to the period of study, where we assume that at the first time 0 there is already an ongoing outbreak (a small number of infectious individuals) and at the last time *T* the study ends. Here the time unit is one day. In the sequel, to refer to the discrete-time interval between any $t_{l}$ and $t_{m}$, we use $\left[t_{l}:t_{m}\right]$ with the convention that the interval is empty when $t_{l}>t_{m}$.

Although the stochastic mechanism of the social network evolution over time is not of primary interest here, the sequence $G_{\left[0:T\right]}$ is however viewed as a realization of a dynamic random network model over the time-period  [ 0 : *T* ]  (see [34]). As previously mentioned, it is reasonable to consider that the social structure is partially random (except the sub-graph corresponding to household interactions), and hence that some individual and edge attributes are governed by some specific probability distributions.

*Infectious disease spreading on the network*

Here, we consider an individual-based SIR dynamic spreading on the underlying social network. The possible individual statuses are only Susceptible (*S*), Infected (*I*) and Removed (*R*), and the only possible status evolution over time are *S* → *I* and *I* → *R*, where *R* considered as an absorbing state. We denote by $X_{t}^{i}\in \{S,I,R\}$ the random variable corresponding to the status of individual *i* at time *t*.

While the dynamic social network is represented by undirected graphs, the transmission of the infectious disease is directed along an edge from an infectious individual (*source* or *donor*) to a susceptible one (*recipient*). We consider that the transmission probability depends on the infection time of the source, as well as on both individual and interaction attributes *A* and *C*. For any time *t* ≥ 0, and any two distinct individuals *i* and *j* in *V*, seen respectively as possible source and recipient, recall that $a^{i},a^{j}\in A$ denote the individual attributes, and $c_{t}^{ij}\in C$ the characteristics of the interaction. In addition, we consider another attribute related to the disease spreading, namely $b_{t}^{i}\in B$, which corresponds to the type of symptoms the individual *i* manifests at time *t*. We have in mind that the individual *i* could be for example asymptomatic, mild, or severe, and the parameter $b_{t}^{i}$ allows us to establish how the severity of the symptoms influences the probability of transmission of *i*. Finally, we denote by $\tau_{I}^{i}$ and $\tau_{R}^{i}$ the not observed random variables representing the infection and removal times of individual *i*, taking values in  [ 0 : *t* ] ∪ { + *∞* } , where for convenience we set $\tau_{I}^{i}=\tau_{R}^{i}=\infty$ if *i* has not yet been infected. We suppose that the transmission probability that the individual *i* infects *j* at *t* depends on all the above quantities (parameters and random variables). Hence, we denote it by $\lambda_{a^{i},a^{j},t,b_{t}^{i},\tau_{I}^{i},\tau_{R}^{i},c_{t}^{ij}}^{i\to j}$ and for the sake of simplicity we use the following simplified notation,


$$\begin{array}{lll} \lambda_{a^{i},a^{j},t,b_{t}^{i},\tau_{I}^{i},\tau_{R}^{i},c_{t}^{ij}}^{i\to j}\equiv \lambda_{\tau_{I}^{i},t}^{i\to j}. & \; & \end{array}$$

Notice that


$$\begin{array}{ccc} \lambda_{\tau_{I}^{i},t}^{i\to j}=0\;\text{if}\;\left(i,j\right)\notin E_{t}\;\text{or}\;t\leq \tau_{I}^{i}\;\text{or}\;t\geq \tau_{R}^{i}. & & \end{array}$$ (1)

*Observations*

Let us now describe the observation process on which the risk computation relies. We assume that testing individuals for the disease is possible from time *t* = 1, so for any *t* ≥ 1 let us denote by $D_{t}^{+}$, $D_{t}^{-}$ the set of individuals receiving a positive, respectively negative result at time *t*. To focus on the most recent and relevant interactions, we introduce the parameter *ζ* ∈ *ℕ* corresponding to the contact tracing time-frame. Hence, at a given time *t* ≥ 0, the set of observations is provided by the graph of interactions during the recent days  [ *t* − *ζ* : *t* ] , and the set of individuals with a positive and a negative result until *t*–1. This set includes test results, interactions network, and both individual and interaction attributes. Notice that we keep the list of all the individuals detected since the beginning because we consider that after they get the infection, they stay immune for the period of study. In conclusion, the set of observations at *t* is defined as


$$O_{t}^{\zeta }={\left\{G_{l}\right\}}_{l\in [t-\zeta :t]}\cup {\left\{D_{l}^{+}\right\}}_{l\in [1:t-1]}\cup {\left\{D_{l}^{-}\right\}}_{l\in [1:t-1]}.$$

In practice, the spreading of the disease on the network is not available. Unless tested or with reported symptoms, the status and infectiousness of individuals are unknown. In addition, there is some uncertainty in the detection process due to the sensitivity and specificity of the tests, and the co-circulation of other diseases causing similar symptoms. However, here we consider only perfect tests (i.e., test specificity and sensitivity equal to 1) and we assume that all the reported symptoms are a consequence of the disease under study.

In particular, the infection and removal times of individuals are never known even for the detected individuals. These quantities are necessary to compute the probability of transmission from a possibly infectious individual *i* to a presumably susceptible *j* at time *t*, as well as the severity coefficient $b_{t}^{i}$, therefore we approximate them. More precisely, we approximate the probability distributions of $\tau_{I}^{i}$ and $\tau_{R}^{i}$, and for the sake of simplicity, we keep the notations $\tau_{I}^{i}$ and $\tau_{R}^{i}$ to represent the random variables issued from the approximated distributions. Depending on the proposed contact tracing approach, we approximate the distribution of $\tau_{I}^{i}$ by a Dirac measure $\delta_{{\hat{\tau }}_{I}^{i}}$ or a generalized truncated geometric distribution, as seen later in Equations (6) and (7). Similarly, we approximate the distribution of $\tau_{R}^{i}$ by a Dirac distribution $\delta_{{\hat{\tau }}_{R}^{i}}$. For convenience, the quantities ${\hat{\tau }}_{I}^{i}$ and ${\hat{\tau }}_{R}^{i}$ are defined in *ℕ* ∪ { + *∞* }  and serve as estimations of $\tau_{I}^{i}$, $\tau_{R}^{i}$. By default, at time 0, we set ${\hat{\tau }}_{I}^{i}={\hat{\tau }}_{R}^{i}=+\infty$ for all *i* ∈ *V*. We briefly describe two distinct situations that we have at any time *t*.

1. If $i\in D_{t}^{+}$, we update the values of the estimators ${\hat{\tau }}_{I}^{i}$ and ${\hat{\tau }}_{R}^{i}$ to finite values computed from the observations. The details on the definition of the finite candidates for ${\hat{\tau }}_{I}^{i}$ are provided later. We set ${\hat{\tau }}_{R}^{i}={\hat{\tau }}_{I}^{i}+\beta$, where *β* is a positive integer that is chosen greater than the mean duration of infectiousness (*β* = 21 in our simulations).
2. If $i\notin D_{t}^{+}$, the values of ${\hat{\tau }}_{I}^{i}$ and ${\hat{\tau }}_{R}^{i}$ stay equal to their previous values.

We denote by ${\hat{b}}_{t}^{i}$ the estimation of the parameter $b_{t}^{i}$ at time *t*. If $i\notin D_{\left[1:t\right]}^{+}$, we consider ${\hat{b}}_{s}^{i}=\text{“asymptomatic”}$ for 0 ≤ *s* ≤ *t*. On the other hand, if *i* is detected at time *t*, that is $i\in D_{t}^{+}$, we update ${\hat{b}}_{l}^{i}$ as the real value $b_{t}^{i}$ for $l\in [{\hat{\tau }}_{I}^{i}:{\hat{\tau }}_{R}^{i}]$ since we assume that when an individual is detected, the severity of the symptoms experienced by this individual is known.

To keep the trace of the negative test results, we define at any *t* and for any individual *j*, the day $\theta_{t}^{j}$ as the last date before *t* on which *j* receives a negative test result,


θtj= max ⁡  {l∈[1:t]:j∈Dl−}, and by convention max ⁡ ϕ=0.

Hence, only the interactions of *j* that are posterior to $\theta_{t}^{j}$ are considered risky, meaning that, before a negative test result, the probability that *j* has been infected is zero.

### Risk of infection via transmission chains

We propose two approaches to compute the risk, based on two different degrees of interactions. To differentiate both methods, we call them in the sequel $1^{\circ }$contact tracing ($1^{\circ }$CT) and $2^{\circ }$contact tracing ($2^{\circ }$CT). In the first approach, the risk of infection is based on $1^{\circ }$ interactions, while the second proposes a more accurate risk of infection, defined from both $1^{\circ }$ and $2^{\circ }$ interactions.

For any time *t* and any individual $j\notin D_{\left[1:t-1\right]}^{+}$, our aim is to estimate the probability of infection of *j* given the set of observations $O_{t}^{\zeta }$, that is


$$\begin{array}{ccc} \mathbb{P}\left(X_{t}^{j}=I\;|\;O_{t}^{\zeta }\right). & & \end{array}$$ (2)

We introduce a new truncation parameter *γ* ∈ *ℕ*, such that *γ* ≤ *ζ*, corresponding to the infection time-frame of interest. More precisely, for any $j\notin D_{\left[1:t-1\right]}^{+}$, we approximate the probability in (2) by “$R_{\gamma ,\zeta }^{j}\left(t\right)$,” which is defined as the probability of *j* being infected in the interval  [ *t* − *γ* : *t* ]  given the set of observations at time *t*, that is


$$\begin{array}{ccc} R_{\gamma ,\zeta }^{j}\left(t\right)=\mathbb{P}\left(\overset{t}{\underset{l=t-\gamma }{⋃}}\left\{\tau_{I}^{j}=l\right\}\;|\;O_{t}^{\zeta }\right). & & \end{array}$$ (3)

The risk given by Equation (3) can be expressed as the probability for individual *j* of having been infected by any of the possible sources of transmission in the time interval  [ *t* − *γ* : *t* ] , given the observations. Hence, Equation (3) can be rewritten as


$$\begin{array}{ccc} R_{\gamma ,\zeta }^{j}\left(t\right) & =1-\underset{i\in V:i\neq j}{\prod }\mathbb{P}\left(\overset{t}{\underset{l=t-\gamma }{⋂}}\left\{Y_{l}^{ij}=0\right\}\;|\;O_{t}^{\zeta }\right), & \end{array}$$ (4)

where


$$\begin{array}{llll} Y_{l}^{ij} & =\{\begin{array}{c} 1,\text{if}i\text{infects}j\text{at time}l,\\ 0,\text{otherwise. } \end{array}\; & & \; \end{array}$$

Then, for any possible individual *i* considered as a source, we can use the law of total probability with respect to the date of infection of *i*, which leads to


$$\begin{array}{ccc} \mathbb{P}\left(\overset{t}{\underset{l=t-\gamma }{⋂}}\left\{Y_{l}^{ij}=0\right\}\;|\;O_{t}^{\zeta }\right)=\underset{d\in \Delta }{\sum }\mathbb{P}\left(\overset{t}{\underset{l=t-\gamma }{⋂}}\left\{Y_{l}^{ij}=0\right\}\;|\;\tau_{I}^{i}=d,O_{t}^{\zeta }\right)\mathbb{P}\left(\tau_{I}^{i}=d\;|\;O_{t}^{\zeta }\right), & & \end{array}$$ (5)

with *Δ* = [ 1 : *t* − 1 ] ∪ { + *∞* } . By independence of the events ${\left\{Y_{l}^{ij}=0\;|\;\tau_{I}^{i}=d,O_{t}^{\zeta }\right\}}_{l\in [t-\gamma :t]}$ we have


$$\begin{array}{lll} \mathbb{P}\;\left(\;\overset{t}{\underset{l=t-\gamma }{⋂}}\;\;\left\{Y_{l}^{ij}=0\right\}|\tau_{I}^{i}=d,O_{t}^{\zeta }\right)\;=\;\overset{t}{\underset{l=t-\gamma }{\prod }}\;\mathbb{P}\left(Y_{l}^{ij}=0|\tau_{I}^{i}=d,O_{t}^{\zeta }\right), & \; & \end{array}$$

where we assume


$$\begin{array}{llll} Y_{l}^{ij}\;|\;\tau_{I}^{i}=d,O_{t}^{\zeta } & ∼\;\text{Ber}\left(\lambda_{d,l}^{i\to j}⊮\left(l>\theta_{t}^{j}\right)\right).\; & & \; \end{array}$$

The way we model $\tau_{I}^{i}\;|\;O_{t}^{\zeta }$ depends on the contact tracing method under consideration and it is explained in the following sections.

#### Infection risk for $1^{\circ }$ contact tracing.

We describe now how to compute the risk defined by Equation (4) for any *j* such that $j\notin D_{\left[1:t-1\right]}^{+}$ using the $1^{\circ }$CT approach that considers as possible sources of infection only the index cases. At a given time *t*, an individual *i* that has been detected up to *t*, is considered as an *index case* for any time *l* between the respective estimated infection and removal times. Thus, we define the set of index cases at time *l* given the set of observations $O_{t}^{\zeta }$ as


$$\begin{array}{llll} I_{l,t} & =\left\{i\in D_{\left[1:t-1\right]}^{+}:l\in ]{\hat{\tau }}_{I}^{i}:{\hat{\tau }}_{R}^{i}[\right\}.\; & & \; \end{array}$$

For the $1^{\circ }$CT approach, we only take into account the interactions with index cases that occur in the interval  [ *t* − *γ* : *t* ] . Consequently, the set of observations reduces to


$$\begin{array}{llll} O_{t}^{\gamma ,1^{\circ }} & ={\left\{G_{l,t}^{1^{\circ }}\right\}}_{l\in [t-\gamma :t]}\cup {\left\{D_{l}^{+}\right\}}_{l\in [1:t-1]}\cup {\left\{D_{l}^{-}\right\}}_{l\in [1:t-1]}\subset O_{t}^{\zeta },\; & & \; \end{array}$$

where $G_{l,t}^{1^{\circ }}=\left(V_{l,t}^{1^{\circ }},E_{l,t}^{1^{\circ }}\right)\subset G_{l}$, $E_{l,t}^{1^{\circ }}$ corresponds to the set edges $E_{l,t}^{1^{\circ }}$ complemented by their attributes and with $E_{l,t}^{1^{\circ }}$ being composed of the $1^{\circ }$ interactions at *l*, that is


$$\begin{array}{llll} E_{l,t}^{1^{\circ }} & =\left\{(i,j)\in E_{l}:i\in I_{l,t},j\notin D_{\left[1:t-1\right]}^{+}\text{ and }\theta_{t}^{j}<l\right\}.\; & & \; \end{array}$$

In addition, the set $V_{l,t}^{1^{\circ }}$ corresponds to the vertices in $E_{l,t}^{1^{\circ }}$ complemented by their attributes.

As we mentioned before, the time of infection of index cases is inferred from the observations and is defined as


$$\begin{array}{ccc} \mathbb{P}\left(\tau_{I}^{i}=d\;|\;O_{t}^{\zeta }\right)=\mathbb{P}\left(\tau_{I}^{i}=d\;|\;O_{t}^{\gamma ,1^{\circ }}\right)=\delta_{{\hat{\tau }}_{I}^{i}}(d). & & \end{array}$$ (6)

As a consequence, Equation (5) becomes


$$\begin{array}{llll} \mathbb{P}\left(\overset{t}{\underset{l=t-\gamma }{⋂}}Y_{l}^{ij}=0\;|\;O_{t}^{\zeta }\right) & =\mathbb{P}\left(\overset{t}{\underset{l=t-\gamma }{⋂}}Y_{l}^{ij}=0\;|\;\tau_{I}^{i}={\hat{\tau }}_{I}^{i},O_{t}^{\gamma ,1^{\circ }}\right).\; & & \; \end{array}$$

Finally, for the $1^{\circ }$CT method, the risk given by (4) for *j* at *t* is defined as


$$\begin{array}{llll} R_{\gamma }^{j,1^{\circ }}\left(t\right) & =1-\underset{i\in V:i\neq j}{\prod }\overset{t}{\underset{l=t-\gamma }{\prod }}\left(1-\lambda_{{\hat{\tau }}_{I}^{i},l}^{i\to j}⊮\left(l>\theta_{t}^{j}\right)\right).\; & & \; \end{array}$$

Remind that, as considered in Equation (1), $\lambda_{{\hat{\tau }}_{I}^{i},l}^{i\to j}=0$ if ${\hat{\tau }}_{I}^{i}=\infty$.

#### Infection risk for $2^{\circ }$ contact tracing.

Here, we derive the computation of the risk defined by Equation (4) by considering as possible sources of transmission both index cases and $1^{\circ }$ contacts. For any individual *j* such that $j\notin D_{\left[1:t-1\right]}^{+}$, when the possible source is an index case, the risk computation is analogous to the one developed for the $1^{\circ }$ CT method. On the other hand, when the possible source is a $1^{\circ }$ contact *i* (such that $i\notin D_{\left[1:t-1\right]}^{+}$), we use the parameter *ζ* (*ζ* ≥ *γ*) as the time-frame for the infection date of *i*, meaning that it lies in the interval of time  [ *t* − *ζ* : *t* − 1 ] . As a consequence, we are interested in $1^{\circ }$ interactions that occur in  [ *t* − *ζ* : *t* − 1 ]  and in the $2^{\circ }$ interactions that occur after a possible transmission due to a $1^{\circ }$ interaction in the interval of time  [ *t* − *γ* : *t* ] .

Hence, the set of observations $O_{t}^{\zeta }$ reduces to $O_{t}^{\zeta ,\gamma ,2^{\circ }}$ ($O_{t}^{\zeta ,\gamma ,2^{\circ }}\subset O_{t}^{\zeta }$),


$${\left\{G_{l,t}^{1^{\circ }}\right\}}_{l\in [t-\zeta :t]}\cup {\left\{G_{l,t}^{2^{\circ }}\right\}}_{l\in [t-\gamma :t]}\cup {\left\{D_{l}^{+}\right\}}_{l\in [1:t-1]}\cup {\left\{D_{l}^{-}\right\}}_{l\in [1:t-1]},$$

where $G_{l,t}^{1^{\circ }}=\left(V_{l,t}^{1^{\circ }},E_{l,t}^{1^{\circ }}\right)$ is defined in ‘Infection risk for 1°contact tracing’ Section, $G_{l,t}^{2^{\circ }}=\left(V_{l,t}^{2^{\circ }},E_{l,t}^{2^{\circ }}\right)\subset G_{l}$, $E_{l,t}^{2^{\circ }}$ corresponds to the set of edges $E_{l,t}^{2^{\circ }}$ complemented by their attributes, and with $E_{l,t}^{2^{\circ }}$ being composed of the $2^{\circ }$ interactions at *l*, i.e.


$$E_{l,t}^{2^{\circ }}=\left\{(i,j)\in E_{l}:\exists k\in V\text{ s.t. }(k,i)\in \underset{d\in [t-\zeta :l-1]}{⋃}E_{d,t}^{1^{\circ }},j\notin D_{\left[1:t-1\right]}^{+}\text{ and }\theta_{t}^{j}<l\right\}.$$

The set $V_{l,t}^{2^{\circ }}$ is composed of the vertices in $E_{l,t}^{2^{\circ }}$ complemented by their attributes.

Let us now consider an individual *i* that has not been detected up to time *t*–1. We remind that, $\theta_{t}^{i}$ is defined as the last time, before *t*, of a negative result test for *i*. On the other hand, *t* − *ζ* is considered as the first possible time of infection of *i*. Hence, we denote by $\theta_{t,\zeta }^{i}=(\theta_{t}^{i}+1)\;\vee \;(t\;-\;\zeta )$ the first possible time of infection for *i*, where the notation "$t_{1}\;\vee \;t_{2}$" stands for the maximal term between $t_{1}$ and $t_{2}$. Then, to bring together all the possible sources of infections (index cases and others), we model the distribution of the time of infection $\mathbb{P}\left(\tau_{I}^{i}=d\;|\;O_{t}^{\zeta ,\gamma ,2^{\circ }}\right)$ of any individual *i* ∈ *V*, and any time *d* ∈ *Δ* = [ 1 : *t* − 1 ] ∪ { + *∞* }  as follows,


$$\begin{array}{ccc} \delta_{{\hat{\tau }}_{I}^{i}}\left(d\right)⊮\left(i\in D_{\left[1:t-1\right]}^{+}\right)+g_{t,\zeta }^{i}\left(d\right)⊮\left(i\notin D_{\left[1:t-1\right]}^{+}\right), & & \end{array}$$ (7)

where $g_{t,\zeta }^{i}(d)$ is the probability mass function of a truncated generalized geometric in $\left[\theta_{t,\zeta }^{i}:t-1]\cup \{+\infty \right\}$, defined as


$$⊮\left(\theta_{t,\zeta }^{i}\leq d\leq t-1\right)p_{d,t}^{i}\;\overset{d-1}{\underset{l=\theta_{t,\zeta }^{i}}{\prod }}\;\left(1-p_{l,t}^{i}\right)+⊮\left(d=\infty \right)\;\overset{t-1}{\underset{l=\theta_{t,\zeta }^{i}}{\prod }}\;\left(1-p_{l,t}^{i}\right).$$

We denote by $p_{l,t}^{i}$ the probability that *i* gets infected by some index case at time *l*, given the set of observations $O_{t}^{\zeta ,\gamma ,2^{\circ }}$, that is


$$p_{l,t}^{i}=1-\underset{k\in V:k\neq i}{\prod }\mathbb{P}\left(Y_{l}^{ki}=0\;|\;\tau_{I}^{k}={\hat{\tau }}_{I}^{k},O_{t}^{\zeta ,\gamma ,2^{\circ }}\right).$$

For the $2^{\circ }$ CT method, the risk of infection $R_{\gamma ,\zeta }^{j,2^{\circ }}\left(t\right)$ for an individual *j* at *t* is defined as,


$$R_{\gamma ,\zeta }^{j,2^{\circ }}\left(t\right)=1-\;\underset{i\in V:i\neq j}{\prod }\underset{d\in \Delta }{\sum }\overset{t}{\underset{l=t-\gamma }{\prod }}\;\mathbb{P}\;\left(Y_{l}^{ij}=0|\tau_{I}^{i}=d,O_{t}^{\zeta ,\gamma ,2^{\circ }}\;\right)\;\mathbb{P}\;\left(\tau_{I}^{i}=d|O_{t}^{\zeta ,\gamma ,2^{\circ }}\right).$$

## Simulation results

Simulated data come from OpenABM-Covid19 model which code is available on https:// github.com/aleingrosso/OpenABM-Covid19. Our code is available on https://github.com/ gbayolo26 /risk_estimation.

### Simulated data

To test our proposed method on a proper data set, we generate the data using the OpenABM-Covid19 model introduced by [27]. This agent-based model simulates the spread of the COVID-19 disease on a sequence of contact networks representing the daily interactions within a population whose demographic structure is based upon UK census data. This model has several advantages: (1) it is rich enough to mimic a dynamic social contact network at the level of a real country, with possible large population size and a variety of individual information, in particular, the daily interactions between individuals come from three different networks depicting the contacts at home, at work and at random; (2) concerning the disease spreading, several infected statuses are available ranging from asymptomatic to pre-symptomatic statuses to mild or severe symptomatic, where the pre-symptomatic status refers to infectious individuals without symptoms; (3) it has several implementation advantages such as a very fast running time and the fact that new intervention methods, like ours, can be easily integrated into the existing code.

In the OpenABM-Covid19 model, the transmission probability takes into account the infectiousness of the source (day of infection, disease severity according to the status), the susceptibility of the recipient based on the age group, and the place where the interactions occur, putting more weight to the household interactions than the others. Indeed, the transmission probability $\lambda_{\tau_{I}^{i},t}^{i\to j}$ that *i* infects *j* at time *t* is defined as


$$\begin{array}{ccc} 1-\text{exp}\;\left(\;-Lf_{A}(a^{j})f_{B}(b_{t}^{i})f_{C}(c_{t}^{ij})\int_{t-\tau_{I}^{i}-1}^{t-\tau_{I}^{i}}f_{\Gamma }(u;\mu ,\sigma^{2})du\;\right), & & \end{array}$$ (8)

where

1. $f_{\Gamma }$ accounts for the varying infectiousness over the course of the disease, and it is chosen as the density function of the Gamma distribution with mean *μ* and standard deviation *σ*,
2. $f_{B}(b_{t}^{i})$ is the severity of the individual *i* considered as a possible source at time *t* (*i* can be susceptible, asymptomatic, pre-mild, mild, pre-severe, severe or removed),
3. $f_{A}(a^{j})$ is the relative susceptibility of the recipient *j*, which depends on the age group of *j*, and is normalized by the mean number of daily interactions by age group,
4. $f_{C}(c_{t}^{ij})$ is the strength of the interaction (if it is at home, work or random) between *i* and *j* at *t*,
5. *L* scales the overall infection rate.

For more details on the OpenABM-Covid19 model, and in particular, on the functions $f_{A}$, $f_{B}$ and $f_{C}$, the interested reader can refer to S1 appendix and [27].

### Intervention strategy

**Fig 1:**
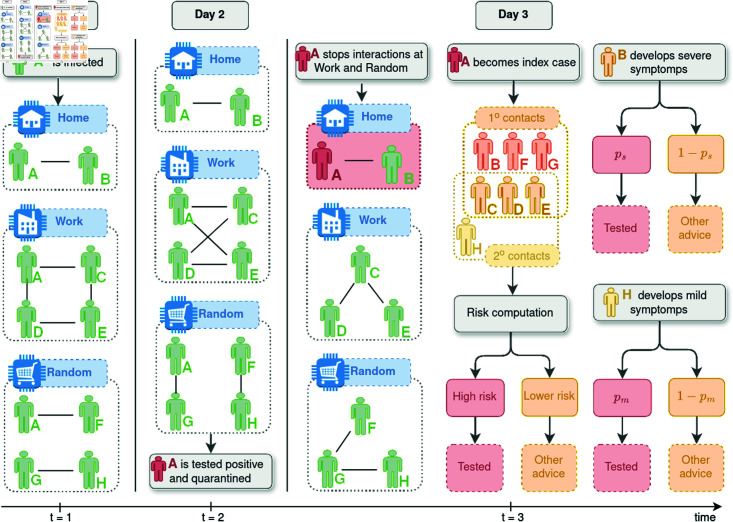
Interactions between individuals over three days. When the individual A is detected, A is quarantined, and their time of infection is estimated. The $2^{\circ }$contact tracing method traces forward the first and second contacts in interaction with A after the estimated time of infection. The risk for these individuals is then computed, and those with the highest risk are selected for testing.

The simulation starts at time *t* = 0 with *N* individuals. At the beginning, all individuals are susceptible (*S*), except for a small number $N_{0}$ of infectious individuals (“patients zero”). Every day, starting from *t* = 1, a proportion $p_{s}$, respectively $p_{m}$, of individuals with newly developed severe and mild symptoms are tested, detected and quarantined. Later, at a fixed date $t_{0}$ in  [ 1 : *T* ] , the intervention based on the risk calculation starts, and it is carried out daily until the mitigation of the epidemic or the end of the study. At any $t\geq t_{0}$, the intervention strategy based on the $2^{\circ }$CT method consists of tracing $1^{\circ }$ and $2^{\circ }$contacts, computing their risk of infection, and ranking them according to their risk values. See Fig 1 for an illustration of how the proposed $2^{\circ }$CT method works for a simple scenario of three days. Then, the first *η* individuals in the ranking are tested, and the newly detected ones become index cases and are quarantined. The default quarantine protocol stops the interactions in the occupation and random network, but those within the household are maintained. On a given day, it may happen that the number of traced individuals is smaller than *η*, in which case we randomly select and test individuals among those who have not been detected, until reaching the number of *η* daily available tests; for those detected, we set their time of infection at *γ* days prior to their detection (since we do not have information to infer their time of infection from previous contacts with detected individuals). As already mentioned, the tests are assumed to be perfect, and we suppose that the test results are available the same day on which the tests are performed.

Notice that the $1^{\circ }$CT method is a particular case of the $2^{\circ }$CT method, and hence, the intervention related to the $1^{\circ }$CT method is analogous to the one for the $2^{\circ }$CT method, except that only the $1^{\circ }$contacts are traced and ranked. The implementation of these methods could be automated via contact tracing applications (DCT), when this tool is available, as follows: (1) data collection, namely index cases record and share their contact data anonymously, $1^{\circ }$ contacts are traced and their subsequent contacts ($2^{\circ }$ contacts) are identified; (2) risk calculation by the DCT app for each traced individual using the predefined risk formula, based on their exposure to index cases; (3) risk-based ranking and notification, meaning that individuals are ranked according to their risk values, and those with the highest risks are prioritized for testing and subsequent quarantine; and (4) real-time updates by the app of the risk scores as new cases are reported or additional contacts are traced.

To compute the risk of infection for the $1^{\circ }$CT and $2^{\circ }$CT methods, we use the transmission probability considered in [27] and defined by Equation (8), with ${\hat{b}}_{t}^{i}$ and ${\hat{\tau }}_{I}^{i}$ in place of the true values $b_{t}^{i}$ and $\tau_{I}^{i}$. Due to the way the infectiousness is modeled, we have that $\lambda_{\tau ,t}^{i\to j}\approx 0$ if *t*  −  *τ* ≥ 15. The latter, combined with a significant gain of the computational cost of our method, leads us to reduce the set of index cases as follows


$$\begin{array}{lll} I_{l,t}=\left\{i\in V:l\in ]{\hat{\tau }}_{I}^{i}\vee (t-21):{\hat{\tau }}_{R}^{i}[\right\}. & \; & \end{array}$$

### Results

In this section, we present the results obtained using the intervention based on the risk, through the simulation of different scenarios. For all the simulations, the propagation of the epidemic is identical until $t_{0}$, while it might change after $t_{0}$, when the intervention method starts, depending on the particular scenario. In the figures presented later, each thin line represents the result obtained for the realization associated with one *seed*, while the thick lines correspond to the average of all the realizations.

#### Estimation of the time of infection.

As seen before, the estimated time of infection of index cases has a direct impact and an indirect impact on the computation of the risk of infection since (1) the probability of transmission depends on it and (2) the selection of the risky interactions relies on it. The estimation of the time of infection is computed on the day of detection of the individual, and it remains constant over time after this day. Remind that, for any individual *j* who has not been detected, we have set ${\hat{\tau }}_{I}^{j}=\infty$.

At any time $t\geq t_{0}$, the individuals are tested because of their symptoms or because they have been traced and selected based on their risk of infection. Among the symptomatic individuals, let us denote by *m* (*m* ∈ *ℕ*) the expected number of days it takes to develop symptoms after the day of infection. In our simulations, we used *m* = 6 as in [27]. Then, if *j* is detected by symptoms at *t* we define the approximation of the time of infection as $\left(t-m)\vee (\theta_{t}^{j}+1\right)$.

For any individual *j* detected by risk at *t*, we define ${\hat{\tau }}_{I}^{j}$ as follows,


$${\hat{\tau }}_{I}^{j}={\hat{\tau }}_{I}^{j,1^{\circ }}⊮\left({\hat{\tau }}_{I}^{j,1^{\circ }}\neq \infty \right)\;+\;{\hat{\tau }}_{I}^{j,2^{\circ }}⊮\left({\hat{\tau }}_{I}^{j,1^{\circ }}=\infty \right),$$

where ${\hat{\tau }}_{I}^{j,1^{\circ }}$ and ${\hat{\tau }}_{I}^{j,2^{\circ }}$ are defined respectively as the minimal time of the $1^{\circ }$ and $2^{\circ }$ interactions of *j*, lying within the time-frame  [ *t* − *γ* : *t* ] , that is


$$\begin{array}{llll} {\hat{\tau }}_{I}^{j,1^{\circ }} & =\min\left\{d\in [t-\gamma :t]:\exists i\in V\text{ s.t. }(i,j)\in \overset{1^{\circ }}{\underset{d,t}{E}}\right\},\; & & \;\\ {\hat{\tau }}_{I}^{j,2^{\circ }} & =\min\left\{d\in [t-\gamma :t]:\exists i\in V\text{ s.t. }(i,j)\in \overset{2^{\circ }}{\underset{d,t}{E}}\right\},\; & & \; \end{array}$$

where, by convention we set *min* ⁡  *ϕ* = *∞*.

To evaluate the effectiveness of the estimator ${\hat{\tau }}_{I}^{j}$ in the mitigation strategy, we propose another estimator ${\hat{\alpha }}_{I}^{j}$ for the individuals detected by risk, which is constant for all index cases provided that they do not have a previous negative test result, that is


$$\begin{array}{lll} {\hat{\alpha }}_{I}^{j}=(t-\gamma \;)\vee (\theta_{t}^{j}+1). & \; & \end{array}$$

**Fig 2:**
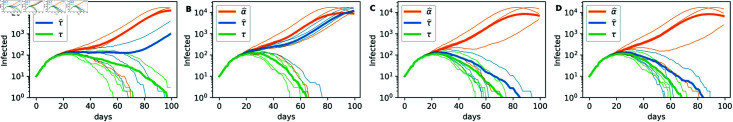
Effect of the estimators ${\hat{\tau }}_{I}^{i}$ (blue), ${\hat{\alpha }}_{I}^{i}$ (orange), and the real time of infection $\tau_{I}^{i}$ (green). (A) The $1^{\circ }$CT method with parameter *γ* = 6. (B) $2^{\circ }$CT with *γ* = 6 and *ζ* = 7. (C) $2^{\circ }$CT with *γ* = 6 and *ζ* = 8. (D) $2^{\circ }$CT with *γ* = 6 and *ζ* = 9. The figures illustrate the impact of these methods on the spread of the epidemic, displaying the number of infectious individuals over *T* = 100 days in a population of size *N* = 50*K*. The intervention begins on day $t_{0}=12$, with $N_{0}=10$ initial patients (patient zero cases), *η* = 125 daily available tests, a proportion $p_{s}=1$ of detected severe symptomatic individuals, and a proportion $p_{m}=0.75$ of detected mild symptomatic individuals.

**Fig 3:**
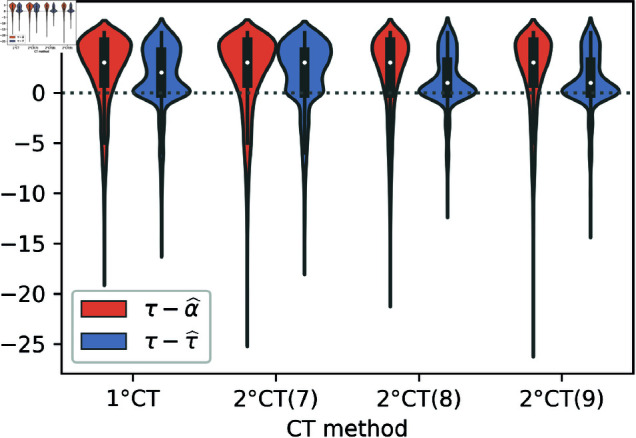
Comparison of infection time estimators. Box plots of the differences $\tau_{I}^{i}\;-\;{\hat{\tau }}_{I}^{i}$ (orange) and $\tau_{I}^{i}\;-\;{\hat{\alpha }}_{I}^{i}$ (blue) for individuals detected by risk using the $1^{\circ }$CT and $2^{\circ }$CT methods, considering the parameter range *ζ* = 7 , 8 , 9 and *γ* = 6. Each box plot was generated using four seeds, with parameters *T* = 100, *N* = 50*K*, $t_{0}=12$, $N_{0}=10$, *η* = 125, $p_{S}=1$, and $p_{m}=0.75$.

To compare the effect of ${\hat{\tau }}_{I}^{i}$, ${\hat{\alpha }}_{I}^{i}$, and $\tau_{I}^{i}$ (the real infection time of *i*), on the mitigation of the epidemic, we simulate the same intervention strategy with these three different times of infection and for different sets of parameter values. We depict in Fig 2 the number of infectious individuals in logarithmic scale through time, and for the same simulated trajectories, we display in Fig 3 the box-plots of the empirical distribution of the differences $\tau_{I}^{i}\;-\;{\hat{\alpha }}_{I}^{i}$ and $\tau_{I}^{i}\;-\;{\hat{\tau }}_{I}^{i}$.

The results in Fig 2 show that, for a broad range of parameter values, the use of the estimated infection time ${\hat{\tau }}_{I}^{i}$ is more effective in the mitigation of the epidemic than the constant estimator ${\hat{\alpha }}_{I}^{i}$. This improvement is more pronounced in panels C and D, in which ${\hat{\tau }}_{I}^{i}$ provides results that are almost as good as the ones obtained with $\tau_{I}^{i}$, the true time of infection. If we take a look at the corresponding box-plots in Fig 3, we can see that the empirical distribution of $\tau_{I}^{i}\;-\;{\hat{\tau }}_{I}^{i}$ is more concentrated around the empirical median, and hence, has less variability, than the one of $\tau_{I}^{i}\;-\;{\hat{\alpha }}_{I}^{i}$. Moreover, for *ζ* = 8 , 9, the empirical median of $\tau_{I}^{i}\;-\;{\hat{\tau }}_{I}^{i}$ is much closer to zero than the one of $\tau_{I}^{i}\;-\;{\hat{\alpha }}_{I}^{i}$. In these latter cases, the good performance of the estimator ${\hat{\tau }}_{I}^{i}$ allows reaching the mitigation of the epidemic faster than the constant estimator (see panels C–D in Fig 2).

#### Time-frame for the time of infection.

In a context of limited resources, providing an effective strategy for mitigating an epidemic goes through the detection at an early stage of the most likely infected individuals. Indeed, the detection of the latter before they become highly infectious is preferable. Hence, tuning the parameter *γ*, which corresponds to the considered infection time-frame for individuals at risk, plays a key role in providing an effective strategy for the mitigation of the epidemic. There is a trade-off between large and small values of *γ*. For large values of *γ*, one expects to detect more individuals since, by construction, the set of observations increases with time. On the other hand, small values of *γ* allow us to concentrate the efforts on the more recently infected individuals, before they propagate the disease, and discard those individuals that were infected a long time ago.

**Fig 4:**
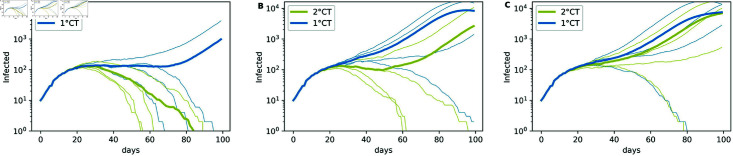
Effect of *γ* on epidemic spread. Effect of *γ* on epidemic spread for strategies $1^{\circ }$CT (blue) and $2^{\circ }$CT (yellow) when *ζ* = *γ* + 3, with (A) *γ* = 6, (B) *γ* = 10, and (C) *γ* = 14. Each plot was generated using four seeds, with parameters *T* = 100, *N* = 50*K*, $t_{0}=12$, $N_{0}=10$, *η* = 125, $p_{S}=1$, and $p_{m}=0.75$.

**Fig 5:**
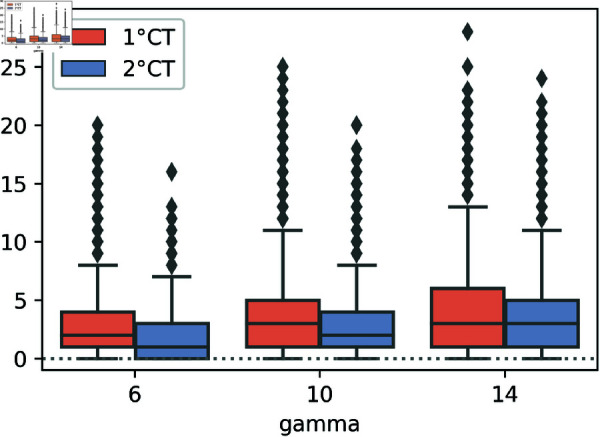
Box plots of infection and detection time differences. Box plots of the differences between the time of infection and the time of detection for individuals detected by risk using the $1^{\circ }$CT and $2^{\circ }$CT methods, with parameter values *γ* = 6 , 10 , 14, and for $2^{\circ }$CT with *ζ* = *γ* + 3. The simulations were performed with *T* = 100, *N* = 50*K*, $t_{0}=12$, $N_{0}=10$, *η* = 125, $p_{S}=1$, and $p_{m}=0.75$, using four different seeds.

In Fig 4, we study the impact of the values of *γ* on the mitigation of the epidemic for $1^{\circ }$CT and $2^{\circ }$CT methods, showing the number of infectious individuals in logarithmic scale through time. For these simulations we keep *ζ* constant with respect to *γ* (the choice of the value of the parameter *ζ* is further discussed in ‘Comparison with other ranking methods’ Section. In Fig 5, we focus on the effect of *γ* on the early or late detection of the individuals by displaying the box-plots of the difference between the time of detection and the time of infection for the individuals detected by risk. Each box-plot in Fig 5 has been built from the same simulations presented in the Fig 4.

Fig 5 shows that, for both methods, the detection of infected individuals is faster with a small value of *γ*; this enables a faster progression of the epidemic mitigation as seen in Fig 4. From both figures, we conclude that taking *γ* = 6 in the two methods makes them effective in detecting and quarantining individuals at an early stage of infection and improves the allocation of resources.

#### Probability of transmission.

The probability of transmission plays also a role in the ability of the contact tracing strategy to mitigate the epidemic. For the $1^{\circ }$CT and $2^{\circ }$CT methods, the probability of transmission is involved in the risk of infection calculated at *t* for an individual *j* and depends directly on the estimation of the time of infection (see Equation (8)).

**Fig 6:**
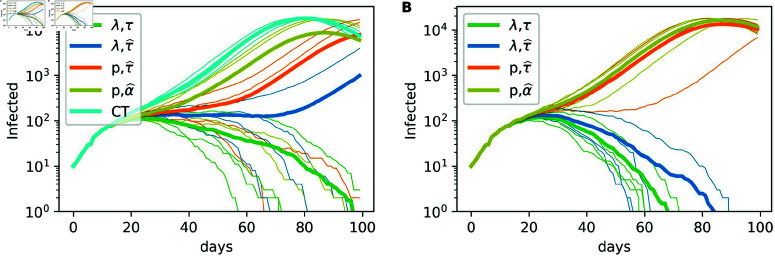
Effect of the transmission probability function on epidemic spread. Effect of the transmission probability function on the spreading of the epidemic for the (A) $1^{\circ }$CT and (B) $2^{\circ }$CT methods. Each plot was generated with *T* = 100, *N* = 50*K*, $t_{0}=12$, $N_{0}=10$, *η* = 125, $p_{m}=0.75$, $p_{S}=1$, *γ* = 6, and for $2^{\circ }$CT with *ζ* = 9.

Here we compare the $1^{\circ }$CT and $2^{\circ }$CT methods for a range of values of the pair “probability of transmission and real or estimated time of infection,” i.e., for $\left(\lambda_{\tau_{I}^{i}}^{i\to j},\tau_{I}^{i}\right)$, $\left(\lambda_{{\hat{\tau }}_{I}^{i}}^{i\to j},{\hat{\tau }}_{I}^{i}\right)$, $\left(p,{\hat{\tau }}_{I}^{i}\right)$ and $\left(p,{\hat{\alpha }}_{I}^{i}\right)$, where $\lambda_{{\hat{\tau }}_{I}^{i}}^{i\to j}$ is defined by Equation (8), ${\hat{\tau }}_{I}^{i}$, ${\hat{\alpha }}_{I}^{i}$ are defined later, $\tau_{I}^{i}$ is the true infection time of *i* and *p* = 1 ∕ 2 is a constant. For all of these pairs, Fig 6 depicts the number of infectious individuals in logarithmic scale through time, for *γ* = 6 and *ζ* = 9.

Both panels in Fig 6 show that the results given by the pair $\left(p,{\hat{\tau }}_{I}^{i}\right)$ (in orange) are much better than the ones obtained with $\left(p,{\hat{\alpha }}_{I}^{i}\right)$ (in yellow), confirming that it is crucial to accurately estimate the infection time. Moreover, the probability of transmission that depends on the individual and interaction attributes (in dark blue) considerably improves the results compared with the choice of a constant probability of transmission.

In Fig 6A, the results provided by the CT method are included; the CT method consists of ranking individuals according to their number of interactions with detected individuals in the time-frame  [ *t* − *γ* : *t* ] . It is important to highlight that the CT method differs from the $1^{\circ }$CT method with $\left(p,{\hat{\alpha }}_{I}^{i}\right)$, since in the CT method the dates of the negative test results are not taken into account. Fig 6A shows that including the information on negative test results has a positive effect on the mitigation of the epidemic.

#### Comparison with other ranking methods.

To evaluate the efficiency of the proposed $1^{\circ }$CT and $2^{\circ }$CT methods to mitigate an epidemic, we compare them with three other ranking strategies:

1. Random Selecting (RS): individuals not previously detected are ranked randomly.
2. Contact Tracing (CT): individuals not previously detected are ranked according to their number of interactions with detected individuals in the time-frame  [ *t* − *γ* : *t* ] .
3. Mean-Field (MF): individuals not previously detected are ranked according to the mean-field risk approximation presented in [2].

We fix the parameters *γ* = 6 and *ζ* = 7 , 8 , 9 (indicated in the legend as $2^{\circ }$CT(7), $2^{\circ }$CT(8), $2^{\circ }$CT(9), respectively). The values for the parameters in MF strategy are $\rho_{MF}=5$ and $t_{MF}=10$.

The mean-field procedure depends on two parameters: (1) $\rho_{MF}$, the mean time elapsed between the time of infection and the time of detection and (2) $t_{MF}$, a parameter called *integration time* on the MF method, meaning that given new observations, the probabilities are updated in the interval $\left[t\;-\;t_{MF}:t\right]$. For the following simulations we consider $\rho_{MF}=5$ and $t_{MF}=10$, as in [2]. In S2 appendix, we provide further details about the main differences in the computation of the mean-field risk and the $2^{\circ }$ CT risk.

**Fig 7:**
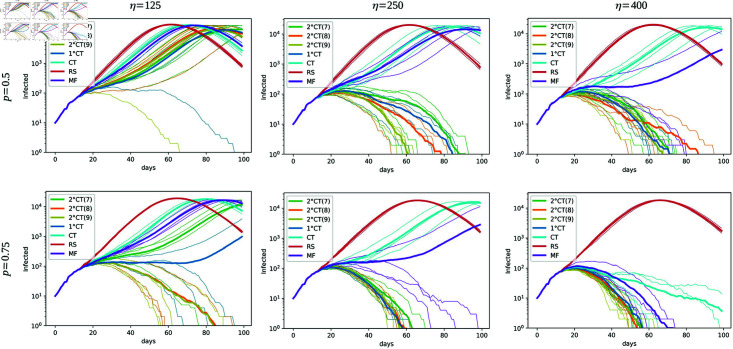
Effect of *η* and $p_{m}$ on epidemic spread. Effect of the parameters *η* (the number of daily available tests, increasing from left to right) and $p_{m}$ (the proportion of daily detected individuals with mild symptoms, increasing from top to bottom) on epidemic spread for the strategies $1^{\circ }$CT, $2^{\circ }$CT, CT, RS, and MF. In all simulations, we consider *T* = 100, *N* = 50*K*, $t_{0}=12$, $N_{0}=10$, and $p_{S}=1$. The estimation of the infection time for the $1^{\circ }$CT and $2^{\circ }$CT strategies is given by ${\hat{\tau }}_{I}^{i}$. We fix the parameters *γ* = 6 and *ζ* = 7 , 8 , 9 (indicated in the legend as $2^{\circ }$CT(7), $2^{\circ }$CT(8), and $2^{\circ }$CT(9), respectively). The parameter values for the MF strategy are $\rho_{MF}=5$ and $t_{MF}=10$.

We compare the five strategies in Fig 7, in which we display the number of infectious individuals in logarithmic scale through time across a broad range of values for the parameters. In particular, we increase the number of daily available tests from the left panels to the right ones, and we increase the proportion of daily detected mild symptomatic individuals from top to bottom. As expected for all strategies, a higher value of $p_{m}$ and/or *η* improves the mitigation of the epidemic in terms of the duration and the total number of infected individuals. The simulations show that our proposed methods ($1^{\circ }$CT and $2^{\circ }$CT) improve considerably the results compared to the MF and the usual CT, which are all better than the RS strategy. The latter does not mitigate the epidemic even with a high number of daily available tests and a high value of $p_{m}$, while the MF and CT methods achieve the mitigation for a large value of *η*. We also study the $2^{\circ }$CT method for different time-frames in which the $1^{\circ }$contact can get infected, that is in  [ *t* − *ζ* : *t* ] , where we consider *ζ* = 7 in green, *ζ* = 8 in orange and *ζ* = 9 in yellow. Fig 7 shows that the results are improved as *ζ* increases. In particular, it should be noticed that the $2^{\circ }$CT method with *ζ* = 8 and *ζ* = 9 gives better results than the $1^{\circ }$CT method. However, the $1^{\circ }$CT method requires less individual information and therefore it is better in terms of privacy restrictions. From these results, a trade-off can arise between getting better results with computationally demanding ($2^{\circ }$CT method) and preserving individual privacy with simpler and faster computation. Indeed, it is worth mentioning that for a high enough number of daily available tests and/or a high enough proportion of mild observed, the methods $1^{\circ }$CT and $2^{\circ }$CT have similar effects on the mitigation of the epidemic; hence in this case, we recommend the use of the $1^{\circ }$CT method than the $2^{\circ }$CT method.

#### Relaxing assumptions.

To better align our model with real-world conditions, we relax several key assumptions, including a fixed delay between symptom onset and testing, perfect tests, and complete quarantine adherence. Previously, we assumed diagnostic tests with 100*%* sensitivity and specificity. While this simplification allowed us to focus on the structural aspects of our model, it does not reflect real-world conditions where diagnostic tests often have lower sensitivity and specificity. To address this, we have expanded our analysis to consider the practical implications of realistic test performance. Specifically, we evaluate how varying levels of sensitivity and specificity impact the effectiveness of our risk-based prioritization strategy. Likewise, we assumed before that all individuals identified for isolation or quarantine adhered fully to these measures. However, in real-world settings, not all individuals comply with quarantine recommendations. In this extended analysis, we relax this assumption by introducing variability in quarantine adoption rates. Specifically, we simulate scenarios where only a fraction of identified individuals adopt quarantine, reflecting different levels of public adherence.

**Mean time to detection based on symptoms**

Initially, the timing between symptom onset and testing was assumed to be fixed; here, we model it explicitly using a geometric distribution to capture the variability in detection times. This distribution reflects real-world scenarios in which testing time is influenced by factors such as severity of symptoms, access to healthcare, and individual behavior. By incorporating this variability, we aim to better capture the stochastic nature of testing delays and their impact on epidemic dynamics. More precisely, we introduce the following parameters,

- $p_{s}$ and $p_{m}$ correspond, for severe and mild individuals respectively, to the daily probabilities of being tested after symptoms onset, which results in a mean delay of $1∕p_{s}$ and $1∕p_{m}$ days between the first signs of the disease and the test.

**Fig 8:**
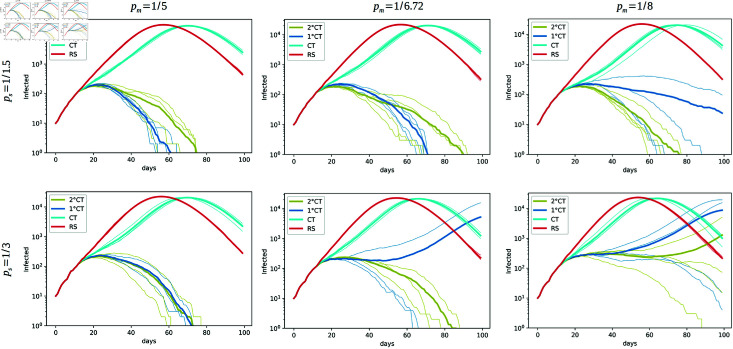
Effect of $p_{m}$ and $p_{s}$ on epidemic spread. Effect of the parameters $p_{m}$ (the daily detection probability of individuals with mild symptoms, decreasing from left to right) and $p_{s}$ (the daily detection probability of individuals with severe symptoms, decreasing from top to bottom) on epidemic spread for the strategies $1^{\circ }$CT, $2^{\circ }$CT, CT, and RS. Each plot is generated from simulations using four seeds, with parameters *T* = 100, *N* = 50*K*, $t_{0}=12$, $N_{0}=10$, and *η* = 400. The estimation of the infection time for the $1^{\circ }$CT and $2^{\circ }$CT strategies is given by ${\hat{\tau }}_{I}^{i}$. We fix the parameters *γ* = 6 and *ζ* = 9.

To evaluate the impact of detection delays for individuals with mild ($p_{m}$) and severe ($p_{s}$) symptoms, we conducted simulations (see Fig 8) in which these parameters were systematically varied. Based on the review provided by [35], the optimal window for conducting RT-PCR testing is between the first and seventh days after symptom onset, with the highest positive result rate seen at a mean of 6.72 days. Accordingly, in our simulation we decreased $p_{m}$ from 1 ∕ 5 (left) to 1 ∕ 8 (right) and we increased $p_{s}$ from 1 ∕ 1 . 5 (top) to 1 ∕ 3 (bottom) to observe how variations in these parameters affect the performance of all the intervention strategies.

Our findings, displayed in Fig 8, indicate that increasing the mean time to detect individuals with mild and severe symptoms (i.e., decreasing $p_{s}$ and $p_{m}$) significantly impacts all strategies by extending the period of undetected transmission. This leads to more secondary infections and a longer epidemic trajectory.

However, when the mean time to detection decreases (as $p_{s}$ and $p_{m}$ increase), the proposed CT risk-based approaches demonstrate resilience in comparison with Random Selection and the usual Contact Tracing. By varying $p_{s}$ and $p_{m}$, our simulations reveal a strong relationship between detection timeliness and epidemic control. Faster detection (higher $p_{s}$ and $p_{m}$) significantly enhances the effectiveness of all strategies. Despite increasing detection delays, the $2^{\circ }$CT method consistently demonstrates superior performance compared to alternative strategies. Reducing the mean time to detection through improved testing accessibility and coverage is crucial. Faster identification and isolation amplify the benefits of risk-based strategies, optimizing the use of limited resources.

These results emphasize the importance of early detection in controlling epidemic spread. Our $1^{\circ }$CT and $2^{\circ }$CT approach remains robust under several detection timelines, but its effectiveness is maximized when detection is prompt, aligning with the important role of efficient public health interventions.

**Sensitivity and specificity**

Here we relax the assumption of perfect diagnostic tests by expanding our simulations to realistic values for sensitivity and specificity, and we evaluate how this impacts our proposed risk-based prioritization strategy.

As mentioned in [36–38], RT-PCR tests, which are considered the gold standard for the diagnosis of COVID-19, have a clinical sensitivity around 90*%* and specificity approximately 95*%*. But the performance of COVID-19 tests is significantly influenced by the severity of symptoms. Indeed, individuals with severe symptoms often have higher viral loads, leading to greater sensitivity in both molecular and antigen tests. Conversely, those who are asymptomatic tend to have lower viral loads, which reduces test sensitivity. For symptomatic individuals, RT-PCR tests demonstrate sensitivity near 100*%* and specificity of approximately 95 . 5*%*, while antigen tests show sensitivity around 96 . 4*%* and specificity close to 98 . 7*%* [37]. It is important to note, as highlighted in [37], that the sensitivity for symptomatic individuals is 100*%* when tests are administered after symptoms onset.

**Fig 9:**
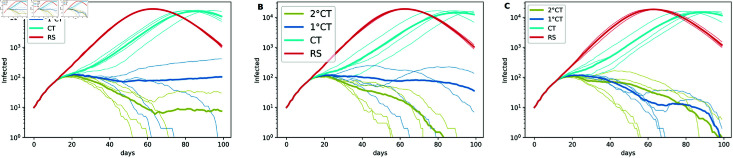
Effect of sensitivity and specificity on epidemic spread. Effect of sensitivity and specificity on epidemic spread for the strategies $1^{\circ }$CT, $2^{\circ }$CT, CT, and RS with (A) *η* = 450, (B) *η* = 500, and (C) *η* = 600. Each plot was generated from simulations using four seeds, with parameters *T* = 100, *N* = 50*K*, $t_{0}=12$, $N_{0}=10$, $p_{s}=1$, and $p_{m}=1∕3$. The estimation of the infection time for the $1^{\circ }$CT and $2^{\circ }$CT strategies is given by ${\hat{\tau }}_{I}^{i}$. We fix the parameters *γ* = 6 and *ζ* = 9.

To address this questions, we conducted simulations with more realistic test characteristics (Fig 9). In our simulations, we modeled different scenarios based on the type of detection and corresponding test characteristics:

- **Testing after symptoms onset:** for individuals tested due to symptoms, we considered perfect sensitivity.
- **Risk-based testing:** For individuals detected through risk-based methods (most of them asymptomatic at the time of testing), we used sensitivity of 90*%* and specificity of 95*%*.

Imperfect sensitivity introduces false negatives, enabling undetected infections to spread, while imperfect specificity generates false positives, potentially misallocating limited testing resources and leading to unnecessary quarantines. Reduced sensitivity slightly undermines the efficiency of identifying and isolating infected individuals, especially when daily testing capacity is constrained, see Fig 9.

Nonetheless, our simulations reveal that even under realistic test performance, the proposed method of $2^{\circ }$ CT remains effective in mitigating epidemic spread. However, achieving this level of mitigation requires increased resources, such as a higher number of daily tests as compared to the same scenario with perfect tests. In contrast, $1^{\circ }$ CT struggles to achieve similar mitigation outcomes under the same conditions.

As expected, realistic test performance slightly reduces the efficiency of our risk-based strategy, but does not compromise its relative advantage over alternative methods such as traditional contact tracing methods and random selection. In settings with limited testing capacity and imperfect diagnostics, the ability to prioritize based on risk might help compensating for the challenges posed by a reduced sensitivity and specificity.

**Quarantine adoption fraction**

To evaluate the effect of varying quarantine adoption rates, we introduce a parameter *q* corresponding to the probability for each individual to adhere to the quarantine recommendations when receiving a positive test result. By varying the values of *q* in our simulations, we explored how changes in the quarantine adoption rate impact the effectiveness of different intervention strategies, particularly our $2^{\circ }$CT risk approach.

**Fig 10:**
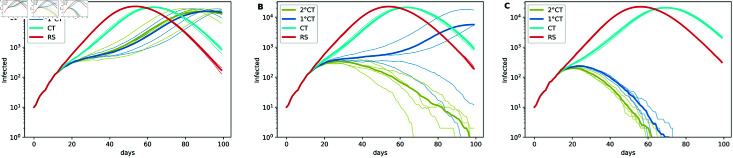
Effect of the quarantine adoption fraction *q* on epidemic spread. Effect of the parameter *q* (the fraction of individuals adopting quarantine), increasing from left to right with (A) *q* = 0 . 9, (B) *q* = 0 . 95, and (C) *q* = 1, on epidemic spread for the strategies $1^{\circ }$CT, $2^{\circ }$CT, CT, and RS. Each plot was generated from simulations using four seeds, with parameters *T* = 100, *N* = 50*K*, $t_{0}=12$, $N_{0}=10$, *η* = 450, $p_{m}=1∕5$, and $p_{s}=1∕3$. The estimation of the infection time for the $1^{\circ }$CT and $2^{\circ }$CT strategies is given by ${\hat{\tau }}_{I}^{i}$. We fix the parameters *γ* = 6 and *ζ* = 9.

Our findings, illustrated in Fig 10, show that reducing quarantine adoption rates significantly affects the performance of all strategies. However, the $2^{\circ }$CT risk-based approach demonstrates remarkable resilience when *q* remains greater than 0.95. Under these conditions, our method continues to outperform naive approaches by prioritizing high-risk individuals for testing and isolation, effectively mitigating epidemic spread despite partial quarantine adoption.

As *q* decreases below 0.95, the overall effectiveness of all strategies diminishes, highlighting the critical role of public adherence to quarantine measures. Lower adoption rates exacerbate the spread of infection, even when $1^{\circ }$CT and $2^{\circ }$CT ranking methods are employed.

These results underscore the necessity of both strategic prioritization and high levels of public cooperation to achieve meaningful epidemic control. The incorporation of variable quarantine adoption rates into our simulations provides a realistic assessment of the challenges and opportunities associated with public health interventions in real-world scenarios.

#### Role of super-spreaders.

Super-spreaders, who infect a disproportionately high number of secondary cases, play a critical role in epidemic dynamics, see [39,40]. Below, we discuss how this phenomenon is considered in our model and provide evidence based on our simulations.

Our method indirectly accounts for super-spreaders by leveraging recent detections of infected individuals and promptly isolating them to decrease their number of contacts within the network structure. Specifically, the risk-based prioritization ranks individuals not only by direct connections to detected cases but also through indirect (up to two-step) transmission pathways. This approach naturally assigns higher priority to highly connected individuals, which are potential super-spreaders.

**Fig 11:**
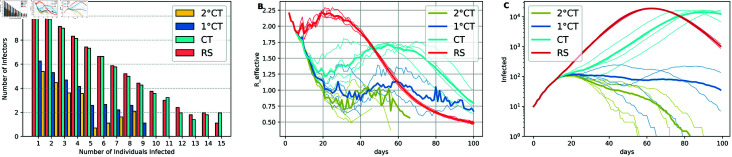
Effect of super-spreaders on ranking methods. Effect of super-spreaders on the ranking methods $1^{\circ }$CT, $2^{\circ }$CT, CT, and RS based on (A) the frequency of secondary infections caused by index cases, (B) the effective reproduction number over time ($R_{\text{effective}}$), and (C) the number of infectious individuals over time. Each plot was generated from simulations using four seeds, with parameters *T* = 100, *N* = 50*K*, $t_{0}=12$, $N_{0}=10$, *η* = 500, $p_{m}=1∕3$, $p_{s}=1$, *q* = 1, sensitivity = 0.9, and specificity = 0.95. The estimation of the infection time for the $1^{\circ }$CT and $2^{\circ }$CT strategies is given by ${\hat{\tau }}_{I}^{i}$. Parameters *γ* = 6 and *ζ* = 9 are fixed across all simulations.

To evaluate the efficiency of the proposed $1^{\circ }$CT and $2^{\circ }$CT methods in identifying and mitigating the impact of super-spreaders, we compare them with other ranking strategies RS and CT, see Fig 11.

We conducted simulations comparing these methods to quantify their ability to address super-spreader dynamics.

- Frequency of secondary infections (Fig 11A):
  We plotted the frequency of the number of infections caused by each infected individual (spreader) across four different seeds, using a logarithmic scale. Results demonstrate that our methods, particularly $2^{\circ }$CT, consistently identified and isolated super-spreaders earlier enough, leading to a significantly lower number of secondary cases as compared to the CT and RS approaches. In contrast, the CT and RS methods allowed super-spreaders to infect more individuals before being isolated, showcasing their relative inefficiency in mitigating the epidemic’s spread.
- Effective reproduction number (Fig 11B):
  We plotted the effective reproduction number ($R_{\text{effective}}$), that is the average number of individuals infected by active infectors at each time step *t*. This quantity plays a crucial role in understanding the influence of super-spreaders on epidemic dynamics. When super-spreaders are active, $R_{\text{effective}}$ tends to be higher due to their ability to amplify the spread of infection through their extensive contact networks. If super-spreader events are not identified and mitigated, $R_{\text{effective}}$ may remain above the critical threshold of 1, allowing the epidemic to grow exponentially. Effective interventions, such as the proposed prioritization of high-risk individuals, can reduce the impact of super-spreaders by quickly isolating them and breaking chains of transmission.
- Number of infectious individuals over time (Fig 11C):
  The number of infectious individuals is plotted over time, also on a logarithmic scale, for the same simulations corresponding to the two previous panels. As expected, better detection of individuals at increased risk of transmission allows more efficient mitigation of the epidemic.

## Discussion

In this paper, we have introduced a method for the computation of the probability of infection of individuals in interaction, based on forward contact tracing, that considers at risk not only the direct contacts of detected individuals but also their subsequent contacts. We have called our method second-degree contact tracing ($2^{\circ }$CT). The proposed method consists of estimating the individual infection risk by considering all possible chains of transmission up to second-degree contacts, coming from index cases. We propose a mitigation strategy that involves using the risk approximation to rank individuals and allocate the limited number of daily available tests accordingly. We have evaluated interventions based on our risk ranking through simulations of a fairly realistic agent-based model calibrated for COVID-19 epidemic outbreak (the Oxford OpenABM-Covid19 model). We have considered different scenarios to study the role of key quantities such as the number of daily available tests, the contact tracing time-window, the transmission probability per contact (constant versus depending on multiple factors), and the age since infection (for varying infectiousness). We found that, when there is a limited number of daily tests available, our method is capable of mitigating the propagation more efficiently than random selection, than the usual contact tracing (ranking according to the number of contacts with detected individuals), and than some other approaches in the recent literature on the subject. Additionally, our risk computation method can be easily adapted to the mitigation of other transmissible diseases spreading on contact networks.

One of the main difficulties in many forward contact tracing approaches for transmission diseases such as COVID-19, is to know the time of infection of the detected individuals. This quantity is in general not observed since in most cases individuals ignore from whom and when they got infected. However, inferring the time of infection is necessary for at least two reasons: (1) to know from which date the contacts of the detected individual should be traced, (2) to accurately assess the risk of infection in the case of varying transmissibility during the course of the disease. Given these arguments, we have considered age-dependent infectiousness, meaning that the probability of transmission from a source *i* depends on the time since infection of *i*. Moreover, we have proposed an efficient estimation of the time of infection for detected individuals, which is more accurate than considering a constant infection time, as it is often proposed in the literature. The results show how this estimation improves the contact tracing method in terms of the number of infectious individuals through time. Indeed, it allows to achieve in some cases almost as good results as considering the real date of infection.

Our $2^{\circ }$CT method encompasses the first degree contact tracing method, called here $1^{\circ }$CT method. We have found that with a limited number of available tests, the $2^{\circ }$CT method is more effective in the mitigation of an epidemic than the $1^{\circ }$CT method. However, with a large enough number of available daily tests, both $1^{\circ }$CT and $2^{\circ }$CT methods provide similar results; in this case, we recommend the use of the $1^{\circ }$CT method because of a simpler and faster computation and better preserving individual privacy. Besides our results show that the proposed $1^{\circ }$CT and $2^{\circ }$CT methods can be very effective compared with the usual contact tracing, the mean-field risk approximation or the random selection of individuals to test.

By integrating test performance into our simulations and analyses, we strengthen the practical relevance of our method, which is designed to work with real-world constraints such as limited resources, imperfect diagnostics, and partial data. This makes it a robust decision-making tool for epidemic mitigation, even under non-ideal conditions. Future work could explore efficient approximations or scalable computational techniques to integrate test characteristics without incurring prohibitive complexity. Such advancements would enhance the precision of the risk-based method while preserving its practicality for real-world applications.

Despite its advantages, our intervention method has some limitations. Firstly, our risk estimation formula assumes perfect tests. Future work could explore the explicit calculation of the risk, or its efficient approximation, to integrate realistic test sensitivity and specificity. Increasing the population size beyond 50k, up to 100k as in [6], or to 500k as in [2], would be another direction for future work. Another assumption that we intend to include in a forthcoming version of our model is the uncertainty in the list of contacts of the traced individuals, and it would be of interest to study how this impacts the efficacy of the intervention. Likewise, the proposed model is primarily designed for the early stages of an epidemic, when the testing capacity is limited and vaccines are unavailable. Incorporating vaccination status in future extensions could enhance the model’s applicability across various stages of an epidemic, broadening its utility in real-world scenarios. Finally, since several models exists, integrating our risk assessment with other risk approaches could enhance the predictive accuracy of the results, reduce uncertainty in prioritization decisions, and enable more targeted interventions tailored to specific regions, epidemic dynamics, or populations.

## Supporting information

S1 Appendix OpenABM-Covid19 model. (PDF)

S2 Appendix Comparison with mean-field. (PDF)
