# Supplementary material for: Test allocation based on risk of infection from first and second order contact tracing
Source: PLoS One. 2025 Apr 7;20(4):e0320291. doi: 10.1371/journal.pone.0320291 (PMC11975095; doi:10.1371/journal.pone.0320291)
Supplement: S1 Appendix — (PDF) [file pone.0320291.s001.pdf]

Appendix 1. OpenABM-Covid19 model

The OpenABM-Covid19 model is a realistic model for the simulation of the COVID-19 epidemic propagation, see [1]. It mimics the interactions between individuals taking into account patterns from UK population. In this model the interactions between individuals are represented by networks that changes every day, this networks are characterised by three different sub-graphs describing the interactions at home, workplace and random. The disease spreads through the networks considering the epidemiological characteristics of COVID-19. Thus, the model takes into account asymptomatic infections and different status of symptomatic infections including stages of severity. Individuals can develop mild or severe symptoms and start by being in a pre-symptomatic state, in which they are infectious but have no symptoms. Once an individual is recovered the model allows immunity.

For the simulations, we use the version presented in https://github.com/aleingrosso/OpenABM-Covid19, in which our test allocation method and tracing techniques can be efficiently integrated. In the following section, we explain this model in details, in particular the generation process of the individual interactions and the disease transmission, as well as the parameters presented in this version and in consequence in our simulations.

Interactions

In the OpenABM-Covid19 model the undirected graph $\mathcal{G}_t = (\mathcal{V}, \mathcal{E}_t)$ have the following characteristics,

1. $\mathcal{V} = \{(i, a^i) : i \in V \text{ and } a^i \in A\}$ is constant through time and it describes individuals i and their associated age group (a^i), with $V = \{1, ..., N\}$ and $A = \{A_1, A_2 \ldots, A_9\}$ the set of age-groups ordered by decade from " $0-9$ years" to " 80+ years ".

2. $\mathcal{E}_t = \{(i, j, c_t^{ij}) : (i, j) \in E_t \text{ and } c_t^{ij} \in C\}$ is the set of the edges describing the interactions at time t between the corresponding individuals, supplemented by the place in which this interaction occurs. Then, $C = H \cup O \cup U$, with $H = \{H_1, H_2, \ldots, H_K\}$ the set of K distinct households according to UK demographics data; $O = \{O_1, \ldots, O_5\}$ the set of distinct occupation activities (primary school, secondary school, workers, retired and elderly); and U the random interactions (interactions at supermarket, public transport, etc). Every individual is assigned to a single household and a single occupation place. The construction of the membership to each household in H and each occupation place in O, as well as the mean number of daily interactions that individuals have, is dependent on the individual age-group.

   For any $t \geq 0$, if i and j are in interaction at time t, it exists a unique edge $(i, j) \in E_t$. In particular, it is assumed the following:

   (a) if two individuals i and j are in the same **household** H_m for $m \in \{1, 2, \ldots, K\}$, then they are in interaction at all times, i.e.,

   $$\mathbb{P}\left((i, j) \in E_t, c_t^{ij} = H_m \,\middle|\, i \in H_m, j \in H_m\right) = 1, \forall t \geq 0,$$

   and if the two individuals are in different households, then they never interact in the household network, i.e.,

   $$\mathbb{P}\left((i, j) \in E_t, c_t^{ij} \in H \,\middle|\, i \in H_m, j \notin H_m\right) = 0, \forall t \geq 0;$$

   (b) for any $m = 1, \ldots, 5$, a fixed sub-graph G_m represents the **occupations** network associated with the occupation activity O_m; the G_m's are modelled as small-world networks. If two individuals i and j are in interaction in the sub-graph G_m for $m \in \{1, 2, \ldots, 5\}$, then at time t each connection is activated with probability $p = 1/2$, i. e.,

   $$\mathbb{P}\left((i, j) \in E_t, c_t^{ij} = O_m \,\middle|\, (i, j) \in G_m\right) = 1/2, \quad \forall t \geq 0,$$

and if the two individuals are not in interaction in G_m for $m \in \{1, 2, \ldots, 5\}$, then they never interact in the occupation network O_m, i.e.,

$$\mathbb{P}\left((i,j) \in E_t, c_t^{ij} = O_m \,\middle|\, (i,j) \notin G_m\right) = 0, \quad \forall t \geq 0.$$

(c) the **random** interactions are selected uniformly over all the remaining possible interactions, in particular excluding previous connections.

Transmissions

In the OpenABM-Covid19 model, the probability that individual i infects individual j at time t depends on the function f_Γ, which represents the Gamma density function with mean μ ($\mu = 6$) and standard deviation σ ($\sigma = 2.5$). The parameter L scales the overall infection rate, and we fix $L = 5.75$ as in [1]. Additionally, the function $f_A(a^j)$ denotes the susceptibility of recipient j based on their age group, where a^j represents the age group of individual j and is determined by,

$$f_A(a^j) = \begin{cases} 0.71/\overline{I}_1, & \text{if } a^j \in A_1 \\ 0.74/\overline{I}_2, & \text{if } a^j \in A_2 \\ 0.79/\overline{I}_3, & \text{if } a^j \in A_3 \\ 0.87/\overline{I}_4, & \text{if } a^j \in A_4 \\ 0.98/\overline{I}_5, & \text{if } a^j \in A_5 \\ 1.11/\overline{I}_6, & \text{if } a^j \in A_6 \\ 1.26/\overline{I}_7, & \text{if } a^j \in A_7 \\ 1.45/\overline{I}_8, & \text{if } a^j \in A_8 \\ 1.66/\overline{I}_9, & \text{if } a^j \in A_9 \end{cases}$$

where $\overline{I}_k$ is the mean number of daily interactions in the k-th age-group. The infectiousness of the source i at t is given by,

$$f_B(b_t^i) = \begin{cases} 1, & \text{if } b_t^i \text{ is pre-severe or severe} \\ 0.48, & \text{if } b_t^i \text{ is pre-mild or mild} \\ 0.29, & \text{if } b_t^i \text{ is asymptomatic} \\ 0, & \text{otherwise} \end{cases}$$

and the strength of the interaction between i and j at t is defined as,

$$f_C(c_t^{ij}) = \begin{cases} 2, & \text{if } c_t^{ij} \in H \\ 1, & \text{otherwise.} \end{cases}$$

Upon infection, an individual can be asymptomatic or develop mild or severe symptoms depending on her/his age group, see Fig A. If the individual develops symptoms, she/he starts by being in a pre-symptomatic state, in which she/he is infectious but has no symptoms. The $\phi_x(a)$ parameters are the probabilities of transition to a particular state depending on the age group of the individual. The ψ_x parameters are gamma distributed and represent the time taken to make the transition. Once an individual is recovered the model considers immunity. See Fig A for a simplified schema of the possible transitions in the OpenABM-Covid19 model.

For more details on the OpenABM-Covid19 model see https://github.com/aleingrosso/OpenABM-Covid19 and [1].

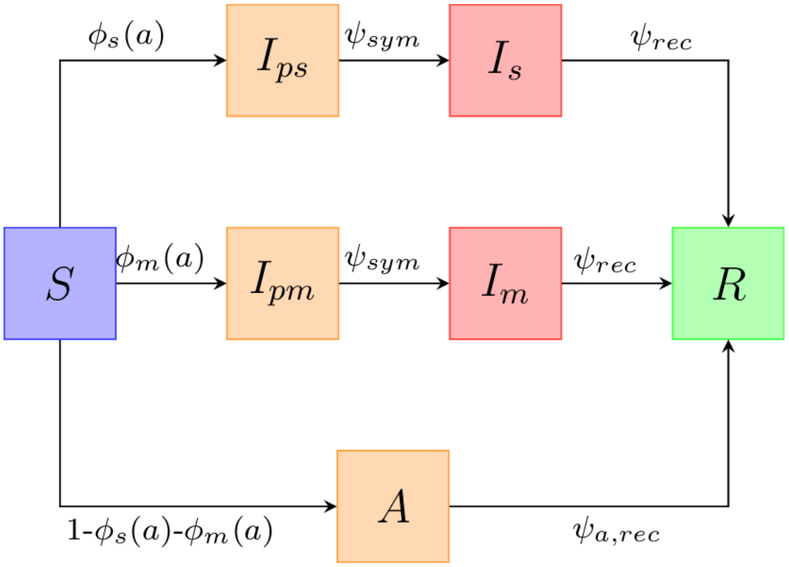

Fig A. Diagram of the possible disease status evolution for an individual.

References

1. Hinch R, Probert WJ, Nurtay A, Kendall M, Wymant C, Hall M, et al. OpenABM-Covid19—An agent-based model for non-pharmaceutical interventions against COVID-19 including contact tracing. PLoS computational biology. 2021;17(7):e1009146.
