# Supplementary material for: Test allocation based on risk of infection from first and second order contact tracing
Source: PLoS One. 2025 Apr 7;20(4):e0320291. doi: 10.1371/journal.pone.0320291 (PMC11975095; doi:10.1371/journal.pone.0320291)
Supplement: S2 Appendix — (PDF) [file pone.0320291.s002.pdf]

S2 Appendix. Comparison with Mean-Field

We illustrate, using the toy example in Fig A, the fundamental differences between our method (2°CT) and the mean-field inference (MF) approach in [1].

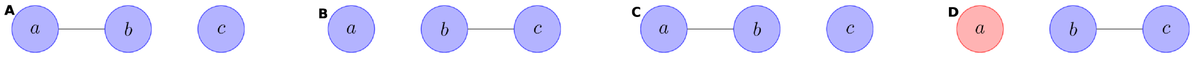

Fig A. Temporal interaction networks over time. Temporal interaction networks at **(A)** $t = 1$, **(B)** $t = 2$, **(C)** $t = 3$, and **(D)** $t = 4$ in a population of $N = 3$. Individual a is detected on day $t = 4$.

First, we point out the difference in computing the risk of infection between the MF and 2°CT methods; indeed, the risk in MF is updated for every individual day-by-day through an iterative process, while the risk in 2°CT is calculated at each day $t \geq t_0$ for only 1°contacts and 2° contacts, by marginalising out over all the possible chains of transmission in the recent past.

Second, we compare the two approaches through the example of 3 individuals a, b, and c, who interact during four days. The individual a is detected at $t = 4$ and is then considered as an index case in $[1:4]$. We assume that a is a patient zero, so the estimated time of infection is $\widehat{\tau}_I^a = 0$. The time of infection for individuals b and c are respectively $\widehat{\tau}_I^b = \infty$, and $\widehat{\tau}_I^c = \infty$, because they have not been detected yet.

Applying the two methods, we determine the risk of infection for b and c at $t = 4$. In Fig B and Fig C, the arrows correspond to the risky interactions considered respectively by the 2°CT and MF methods, to compute the risk of infection of b and c at time $t = 4$. The values below/above the arrows correspond to the times at which these interactions take place.

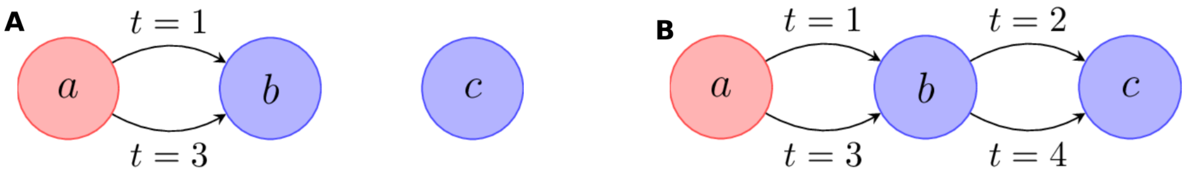

Fig B. Risky interactions considered in the 2°CT method. Risky interactions considered in the 2°CT method to compute: **(A)** the risk of b at time $t = 4$, and **(B)** the risk of c at time $t = 4$.

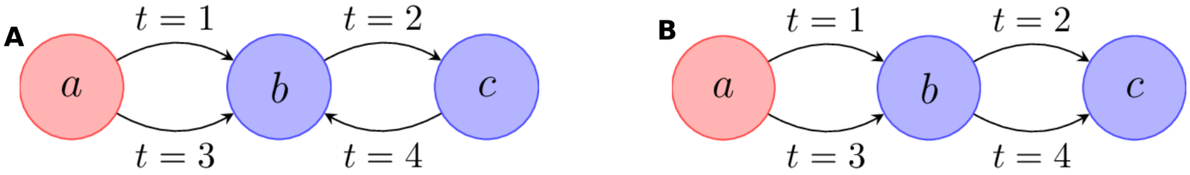

Fig C. Risky interactions considered in the MF method. Risky interactions considered in the MF method to compute: **(A)** the risk of b at time $t = 4$ and **(B)** the risk of c at time $t = 4$.

We apply 2°CT method with $\gamma = \zeta = 4$. At time $t = 4$, b is a 1°contact of a, since they interacted at days $t = 1, 3$. As well, c is 2°contact of a since c interacted with b at days $t = 2, 4$. Hence, for the example presented in Fig. A, the risk of infection for the individual b at time $t = 4$ using the 2°CT method is,

$$
\begin{aligned}
R_{4,4}^{b,2^\circ}(4) &= 1 - \mathbb{P}\left(\bigcap_{l=1}^{4} Y_l^{ab} = 0 \middle| \mathcal{O}_4^{4,4,2^\circ}\right) \mathbb{P}\left(\bigcap_{l=1}^{4} Y_l^{cb} = 0 \middle| \mathcal{O}_4^{4,4,2^\circ}\right) \\
&= 1 - (1 - \lambda_{0,1}^{a \to b})(1 - \lambda_{0,3}^{a \to b})
\end{aligned}
$$

and the risk of infection for the individual c at time $t = 4$ is,

$$R_{4,4}^{c,2^\circ}(4) = 1 - \mathbb{P}\left(\bigcap_{l=1}^{4} Y_l^{ac} = 0 \middle| \mathcal{O}_4^{4,4,2^\circ}\right) \mathbb{P}\left(\bigcap_{l=1}^{4} Y_l^{bc} = 0 \middle| \mathcal{O}_4^{4,4,2^\circ}\right)$$

where $\mathbb{P}\left(\bigcap_{l=1}^{4} Y_l^{ac} = 0 \middle| \mathcal{O}_4^{4,4,2^\circ}\right) = 1$ and,

$$\mathbb{P}\left(\bigcap_{l=1}^{4} Y_l^{bc} = 0 \middle| \mathcal{O}_4^{4,4,2^\circ}\right) = \sum_{d \in \{1,2,3,\infty\}} g_{5,5}^{b}(d) \prod_{l=1}^{4} \left(1 - \lambda_{d,l}^{b \to c}\right).$$

Then, we have,

$$R_{4,4}^{c,2^\circ}(4) = \lambda_{0,1}^{a \to b} \prod_{l \in \{2,4\}} (1 - \lambda_{1,l}^{b \to c}) + \lambda_{0,3}^{a \to b}(1 - \lambda_{0,1}^{a \to b})(1 - \lambda_{3,4}^{b \to c}) + \prod_{l \in \{1,3\}} (1 - \lambda_{0,l}^{a \to b}).$$

For the MF method, we suppose that the time between infection and testing is greater than 4, and we set $t_{MF} = 4$, (t_{MF} stands for the integration time of the MF process). Once a is detected, to compute the MF risk we assume that the initial risk of a is 1, while for b and c it is considered as 0. Therefore, the MF risk of the individual b at time $t = 4$ is

$$\begin{aligned} R_{\mathrm{MF}}^{b}(4) &= 1 - (1 - p_1^{a \to b})(1 - p_3^{a \to b})(1 - p_4^{c \to b} p_2^{b \to c} p_1^{a \to b}) \\ &= 1 - \left(1 - R_{\mathrm{MF}}^{b}(3)\right)\left(1 - p_4^{c \to b} R_{\mathrm{MF}}^{c}(3)\right), \end{aligned}$$

where $p_t^{i \to j}$ is the probability that i infects j at time t, which does not depend on the time of infection of the source i.

The MF risk of the individual c at time $t = 4$ is

$$\begin{aligned} R_{\mathrm{MF}}^{c}(4) &= 1 - \left(1 - p_1^{a \to b} p_2^{b \to c}\right)\left[1 - p_4^{b \to c}\left(1 - \prod_{t \in \{1,3\}} \left(1 - p_t^{a \to b}\right)\right)\right] \\ &= 1 - \left(1 - R_{\mathrm{MF}}^{c}(3)\right)\left(1 - p_4^{b \to c} R_{\mathrm{MF}}^{b}(3)\right). \end{aligned}$$

Notice that in the MF method, the risk is propagated “bidirectionally”, meaning that it is not only “forward” in the direction of the transmission given the observations, as the proposed 2°CT method. Hence, whenever two individuals interact, they interchange and upgrade their risk regardless of where their infection came from, and assuming independence in all possible transmission chains. For example, in Fig A at time $t = 2, 4$, individuals b and c are in contact and interchange their risk. As a consequence, the risk of b, $R_{\mathrm{MF}}^{b}(4)$, is increased by the current risk of c, but the risk carried by c came from individual b. This is what we call a *cycling back* effect, which can cause an artificial increase of the risk for some individuals in the population. Moreover, in some cases it allows the risk of c to be greater than the risk of b, that is $R_{\mathrm{MF}}^{b}(4) \leq R_{\mathrm{MF}}^{c}(4)$. Instead, the 2°CT method avoids this effect and guarantees that, for any possible transmission probability function per interaction, we have that $R_{4,4}^{b,2^\circ}(4) \geq R_{4,4}^{c,2^\circ}(4)$.

References

1. Baker A, Biazzo I, Braunstein A, Catania G, Dall'Asta L, Ingrosso A, et al. Epidemic mitigation by statistical inference from contact tracing data. Proceedings of the National Academy of Sciences. 2021;118(32):e2106548118. doi:10.1073/pnas.2106548118.
